# Health Information Exchange: Contributions from 2023

**DOI:** 10.1055/s-0044-1800742

**Published:** 2025-04-08

**Authors:** Meryl Bloomrosen, Sue S. Feldman

**Affiliations:** 1 American Medical Association, Chicago, IL, USA; 2 Graduate Programs in Health Informatics, Department of Health Services Administration, University of Alabama at Birmingham, Birmingham, AL, USA; 3 Department of Biomedical Informatics and Data Science, Heersink School of Medicine, University of Alabama at Birmingham, Birmingham, AL, USA

**Keywords:** International Medical Informatics Association Yearbook, Health Information Exchange, Health Information, Interoperability, Data Sharing, Health Information Technology, Electronic Health Records

## Abstract

**Objectives** : To summarize the recent literature and research and present a selection of the best papers published online and in print in 2023 related to health information exchange (HIE).

**Methods** : Using Covidence as a screening and analysis tool, a systematic review of the literature was independently conducted by the two section editors. Seven studies emerged as suitable for final IMIA Yearbook consideration.

**Results** : Among the papers reviewed, three major themes emerged: clinical services utilization, continuity of care, and public and population health. These themes represent an increased breadth and depth of HIE application.

**Conclusions** : Review of the literature suggested more studies with the use of data from HIEs, perhaps suggesting increased trust in data accuracy, adequacy, and completeness. The section editors noted the increase in papers from diverse countries describing applications of HIEs suggesting more widespread implementation of HIEs worldwide. As health data standards are developed and adopted globally, this could set the stage for increased international health data exchange. The larger corpus of 2023 literature reviewed resulted in conversations by the section editors on the changing landscape of the expanding, maturing, and innovative use cases for HIEs and HIE data. This landscape bears continued watching in 2024.

## 1. Introduction

Health Information Exchange (HIE) refers to the electronic sharing of a broad range of health-related information among different healthcare organizations and often disparate systems [ 1 , 2 ]. This process of information sharing, which can be facilitated by an organization called an HIE, aims to facilitate access to and retrieval of clinical data to provide safer, more timely, efficient, effective, and equitable patient-centered care [ 1 , 3 4 5 ]. By enabling the seamless exchange of medical information, HIEs can help to reduce the duplication of tests, minimize treatment delays, and enhance clinical decision-making [ 1 , 3 4 5 ]. Furthermore, HIEs can play a crucial role in public health by improving the ability to track and manage diseases and conditions across populations. As healthcare continues to evolve in the digital age, HIEs are becoming increasingly vital in ensuring that healthcare providers have the information they need to deliver high-quality care.

Globally, nations have adopted various approaches and levels of HIE. Country-specific legislative and regulatory policy impacts HIE implementation and governance. There are differences in data collected, governance, scope, financing, levels of data sharing, technical and technological infrastructure, maturity and public policy considerations [ 6 7 8 9 ].

There are several ways to categorize HIE when discussing how HIE contributes to a more connected and efficient health care system to enhance the quality of care, lower the cost of care, and increase care continuity. For example, there are depictions of “types” of exchange: 1) directed exchange, which allows the secure exchange of health-related information between health providers [ 10 ], 2) query-based exchange, which allows providers to search for and retrieve health-related information on specific people for a specific treatment [ 11 ], and 3) consumer-mediated exchange, which puts the patient at the center of the access, management, and sharing of health-related information [ 12 ]. While various forms of these HIEs exist globally, depending on maturity, each of these types of HIE can co-exist in the same technological environment and include the same types of data, but they are more likely to exist in different technical environments that have expanded the traditional definition of an HIE and include different types of data with increased specificity, depending on the use and user. Whereas the traditional perspective of HIE was focused around provider generated exchange (directed exchange and query-based exchange), HIEs are grappling with how to accommodate consumer-mediated exchange [ 12 ]. For example, the survey paper by Dullabh *et al.* illustrates two different mechanisms for consumer-mediated exchange to occur [ 13 ]. It is important to note that, regardless of the mechanism, consumer-mediated exchange involves more than who can see and use what health-related data. Consumer-mediated exchange can also include consumer-generated data, such as that from implantables and wearables. Data from implantables, for example a pacemaker, has an element of data control and therefore may be more trusted than data from wearables, such as a fitness watch [ 14 ]. Wearables are often controlled by external applications with application programming interfaces (APIs) allowing for information to be sent to or accessed by a third party, and because of this third party, consumer-generated data from wearables, while important and accurate may not share the same level of trust as that from implantables.

The 2023 articles that were reviewed showed a greater focus on the *use of data from* HIEs to conduct research and analysis rather than the *actual use of the HIE to impact care* . While the use of data from HIEs to conduct and inform research was not the focus of the final paper selections, this shift could suggest that researchers increasingly believe that the data from HIEs is accurate, adequate, and complete enough to draw conclusions worthy of integration into a learning health system or knowledge health system. We also noted more papers describing the use of HIEs from diverse countries, suggesting that HIE use is maturing worldwide. Global efforts are underway to develop and adopt the International Patient Summary 1 , which is a set of basic patient level, clinical data for use in the case of an unexpected or unscheduled medical situation (e.g. emergency or accident). While this does not suggest any level of interoperability to facilitate global exchange of health-related information, it is encouraging that the potential may exist.

## 2. About the paper Selection

In February 2024, with the assistance of a medical librarian, the co-editors conducted a PubMed and Embase search using MeSH headings, keywords, and synonyms in titles and abstracts with a focus on HIE. The publication year included first online and print publications between January 1, 2023 and December 31, 2023, inclusive. The search strategy is shown in Table 1 .

**Table 1.** Search Strategy

PubMed		Embase
(“Health Information Exchange”[Majr] OR Health-Information-Exchange*[Title/Abstract] OR Medical-Information-Exchange*[Title/Abstract] OR Clinical-information-system* OR clinical-pharmacy-information-system*[Title/Abstract] OR health-information-network*[Title/Abstract] OR health-information-system*[Title/Abstract] OR medical-information-service*[Title/Abstract] OR is-h-med[Title/Abstract]) AND (“2023/01/01”[Date - Publication] : “2023/12/31”[Date - Publication])		‘(‘medical information system'/exp/mj OR ‘health information exchange*’:ab,ti OR ‘medical information exchange*’:ab,ti OR ‘clinical-information-system*or clinical-pharmacy-information-system*’:ab,ti OR ‘health information network*’:ab,ti OR ‘health information system*’:ab,ti OR ‘medical information service*’:ab,ti OR ‘is h med’:ab,ti) AND [2023-2023]/py AND (‘article’/it OR ‘article in press’/it OR ‘review’/it)

**Figure 1.**
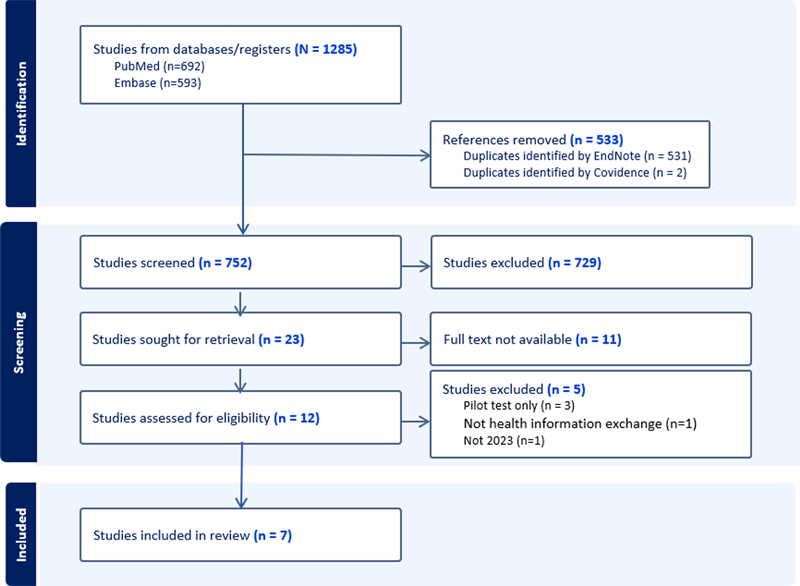
PRISMA diagram of literature search (Generated from Covidence.org)

All studies were imported into EndNote ^®^ for first round deduplication. The remaining studies were imported into Covidence 2 for further deduplication, screening, and analysis. Each of the two section editors independently screened 752 studies. Inclusion differences were reconciled through conversation and then by eventual mutual agreement between the section editors. Of the 12 studies for consideration, five were excluded, resulting in seven studies for final IMIA Yearbook consideration ( Figure 1 ).

A content summary of the best papers ( Table 2 ) can be found in the appendix of this synopsis.

**Table 2.** Selection of best papers for the 2024 IMIA Yearbook of Medical Informatics for the section Health Information Exchange. The articles are listed in alphabetical order of the first author's surname.

Section Health Information Exchange
Aniekwe C, Cuffe K, Audu I, Nalda N, Ibezim B, Nnakwe M, Anazodo T, Dada M, Romano ER, Okoye M, Martin M. Assessing the effect of electronic health information exchange on the completeness and validity of data for measuring viral load testing turnaround time in Nigeria. International Journal of Medical Informatics. 2023 Jun 1;174:105059. https://doi.org/10.1016/j.ijmedinf.2023.105059 Sloan-Aagard C, Glenn J, Nañez J, Crawford SB, Currey JC, Hartmann E. The impact of community health information exchange usage on time to reutilization of hospital services. The Annals of Family Medicine. 2023 Jan 1;21(1):19-26. https://doi.org/10.1370/afm.2903

## 3. Outlook

The seven candidate best papers for 2023 reflected an increase in international HIE usage and more attention to the validity of the data retrieved through an HIE. The use of HIE for public health purposes was noted as a cross-cutting domain where innovative uses of HIE were recognized. Below, we discuss the major themes of the seven papers from 2023 that were candidates for being selected as a “Best Paper for 2024 Yearbook HIE Section”.

### 3.1 Clinical Services Utilization

Three of the seven studies examined the use of an HIE to impact clinical services utilization. A study by Adler-Milstein *et al.* used secondary data sets to identify HIE participation and then looked at the impact of HIE participation on repeat imaging and hospital admission. This study found that HIE participation was not associated with decreased repeat imaging [ 15 ]]. The authors report on hospital admission in terms of admission following a treat-and-release emergency department (ED) visit, which was associated with a lower likelihood of hospital admission and admission following discharge, which was associated with a higher likelihood of admission. A second study examined the availability of patient information and the impact on repeat diagnostics, primarily in oncology patients in The Netherlands [ 16 ]. This study showed that diagnostics were repeated unnecessarily in 15.8% of the study sample. The last of these three studies, by Sloan-Aagard *et al.* , one of the two papers selected as a best paper for the 2024 Yearbook, is one of a few studies that looks at clinical services utilization with HIE use of community primary care physicians [ 17 ]. Often referred to as the “white space” of electronic health record adoption and subsequent HIE connectivity, community-based primary care was largely ignored in the early days of United States (US) regulations incentivizing health data collection, exchange and reporting. As such, there was little motivation for community physicians to be HIE participants. The study by Soan-Aagard *et al.* suggested that querying the HIE had a statistically significant association with reducing ED visits by 53% and a rehospitalization reduction by 61%. It further showed that HIE querying was associated with an increased median time to use ED services. This study is presented in more detail in the appendices of this synopsis.

### 3.2 Continuity of Care

There have been a number of efforts to associate the use of HIE in contributing to care continuity, especially in the mental health space and other chronic care conditions where patients are often “lost” during hand offs [ 18 19 20 ]. As such, this study by Itzhaki *et al.* , details the criticality of HIE awareness among all stakeholders prior to HIE-facilitated information sharing [ 21 ]. While the findings from this study are not new and have been articulated by others [ 22 ], this study is set in Israel where HIEs are less mature than as compared to that in the US and further illustrates global HIE implementation and use.

### 3.3 Public and Population Health

Three of the seven studies considered for the IMIA Yearbook cut across HIE and public or population health illustrating a greater use of HIE in this domain. The first study, conducted by Feldman *et al.* (no relation to the co-editor), examined a partnership between Maryland's HIE, Chesapeake Regional Information System for Our Patients (CRISP), and the Maryland Department of Health to use CRISP as a transport vehicle for positive COVID-19 test results [ 23 ]. The use of CRISP as a transport vehicle facilitated more accurate patient matching and more expeditious contact tracing efforts. While there are other studies detailing the value of near real-time data transmission of COVID-19 test results [ 24 , 25 ], the innovative use of CRISP to do so made a valuable contribution to the HIE literature for public health uses and demonstrated a real-world use case that could prove beneficial in future times of widespread communicable disease situations.

Another innovative use case of HIE for public and population health was for refugees as they entered a military camp in the US (Indiana) from Afghanistan. What made this study unique was the fact that 50% of the 6600 refugees were under 18 years, necessitating an expansion in the traditional public health focus to pediatrics [ 26 ]. This was further complicated by a concurrent surge in COVID-19 cases in Indiana, making this use case one of population *and* public health significance. A centralized refugee inbox was created to facilitate expeditious and secure communications between providers. Additionally, the creation of a primary care identifier that was linked to the camp address, allowed for refugees to have a temporary, yet primary medical home for health data exchange and secure communications, resulting in almost 2700 messages during the 20-week study period. Of those 2700 messages, almost 73% were exchanged with the Children's Hospital. Across all messages, 56% were ED related. It was a bit surprising that only 23% of the messages were related to laboratory and radiology messages. This study illustrated the utility for leveraging existing partnerships and a robust HIE to provide healthcare services for a transient or temporary population.

The last paper to be considered for the IMIA Yearbook, was conducted in Nigeria, and highlighted the effect of data completeness of HIE data on HIV viral load testing turnaround times. This study suggested that data completeness increased from 47% pre-HIE implementation to 67% 6-months post-HIE implementation [ 27 ]. This study was selected for inclusion in the IMIA Yearbook and is therefore presented in more detail in the appendices.

## 4. Conclusion

Studies on HIE published in 2023, of which seven are summarized here, suggested increased breadth and depth in the use of data *from* HIEs and the *use of* HIEs to impact clinical services utilization, continuity of care, and public and population health. The HIE section editors' note that can be viewed as a follow on to finding from the 2023 IMIA Yearbook showing HIE being used in more diverse settings [ 3 ]. It is further noted that these studies set the stage for more innovation around the use of HIEs as a transport mechanism to improve care across a variety of unconventional settings. The survey paper also sets the stage for better integration of HIEs and consumer-mediated HIE and represents an area ripe for more research in this area, especially in the use of consumer-generated health information in clinical decision-making.

## Footnotes

1
https://international-patient-summary.net/

2
https://www.covidence.org
